# Antibiotic Toxicity and Absorption in Zebrafish Using Liquid Chromatography-Tandem Mass Spectrometry

**DOI:** 10.1371/journal.pone.0124805

**Published:** 2015-05-04

**Authors:** Fan Zhang, Wei Qin, Jing-Pu Zhang, Chang-Qin Hu

**Affiliations:** 1 National Institutes for Food and Drug Control, Graduate School of Peking Union Medical College, Beijing, China; 2 Institute of Medicinal Biotechnology, Chinese Academy of Medical Sciences & Graduate School of Peking Union Medical College, Beijing, China; Institut Curie, FRANCE

## Abstract

Evaluation of drug toxicity is necessary for drug safety, but in vivo drug absorption is varied; therefore, a rapid, sensitive and reliable method for measuring drugs is needed. Zebrafish are acceptable drug toxicity screening models; we used these animals with a liquid chromatography-tandem mass spectrometry (LC-MS/MS) method in a multiple reaction monitoring mode to quantify drug uptake in zebrafish to better estimate drug toxicity. Analytes were recovered from zebrafish homogenate by collecting supernatant. Measurements were confirmed for drugs in the range of 10–1,000 ng/mL. Four antibiotics with different polarities were tested to explore any correlation of drug polarity, absorption, and toxicity. Zebrafish at 3 days post-fertilization (dpf) absorbed more drug than those at 6 h post-fertilization (hpf), and different developmental periods appeared to be differentially sensitive to the same compound. By observing abnormal embryos and LD_50_ values, zebrafish embryos at 6 hpf were considered to be suitable for evaluating embryotoxicity. Also, larvae at 3 dpf were adapted to measure acute drug toxicity in adult mammals. Thus, we can exploit zebrafish to study drug toxicity and can reliably quantify drug uptake with LC-MS/MS. This approach will be helpful for future studies of toxicology in zebrafish.

## Introduction

Drug toxicity is a chief reason for drug withdrawal from the market, and toxicity limits clinical use of many compounds [1,2]. Studies suggest that $802 million and an average of 7 years are needed to develop a new compound and that only 1 in 5,000 candidate compounds actually enters clinical trials [3]. Thus, accurate toxicity assessment can increase the chances that a drug will reach consumers. Recently, zebrafish (Danio rerio) have been used in genetics, drug discovery, compound optimization, toxicology, and drug safety screening [4–7]. Zebrafish genes have homologous to human genes of approximate 70%, and partly zebrafish protein sequences, such as app, psen and pen2, also show over 70% homology to human corresponding proteins [8–9]; and they possess similarities with respect to nervous and cardiovascular systems, liver, pancreas, intestine, gallbladder, and certain metabolic pathways. Also, zebrafish have cytochrome P450 enzymes and nuclear receptors [10] similar to mammals, so this species has been utilized as a model for pharmacology and toxicology studies [11,12]. It is thought that zebrafish may offer alternative models to more expensive, restrictive, and time-consuming rodent models [13,14]. Zebrafish models can be used to assess organ toxicity and mechanisms behind such toxicity by mimicking human genetic, phenotypic, and ethological characteristics [15,16]. Also, drug impurities can be evaluated with zebrafish embryo toxicity tests [17,18]. Many studies suggest that drug toxicity screening with zebrafish is informative [19–24], and that zebrafish can be used to predict some drug responses and toxicity in humans [25]. Also, compound absorption as measured in zebrafish is difficult to extrapolate to humans to estimate toxicity. For example, erythromycin and D-sotalol which cause QT prolongation in mammals are poorly absorbed in zebrafish and did not alter zebrafish larval heart rates. Thus, no apparent toxicity was evident [26]. Similarly, sodium valproate, which can cause acute liver failure in humans, did not produce hepatotoxic effects in adult zebrafish likely because the compound was not sufficiently absorbed to induce liver abnormalities. In contrast, zebrafish larvae absorbed sodium valproate, and liver abnormalities were observed [27,28]. Therefore, confirming and quantifying compound uptake in zebrafish is needed to avoid potentially false-positive or-negative results in zebrafish toxicity tests [29].

Few studies are available to assess toxicity and quantification of compound absorption in zebrafish, so we tested four drugs in zebrafish across different developmental stages. Specifically, we studied antibiotic uptake and assessed toxicity at critical embryonic stages with LC-MS/MS and microscopic observation of abnormal embryos. We also measured LD_50_ values for each compound.

## Materials and Methods

### 1.1 Reagents and solution preparation

Ceftazidime, minocycline, cefotaxime, and netilmicin used in the experiments were national reference substances obtained from the National Institutes for Food and Drug Control. Using LogP to estimate membrane permeability, we chose four compounds with different polarities to broaden the applicability of the analytical method (LogP of ceftazidime: 0.4, minocycline: -0.6, cefotaxime: -1.4, and netilmicin: -4.3). Stock solutions (1.0 mg/mL) each were prepared from dissolved drug powder in methanol with formic acid (0.1%, v/v) and stored at 4°C for HPLC optimization. All reagents used were of analytical quality. Methanol and acetonitrile for HPLC were provided by Thermo Fisher Company (Waltham, MA). Deionized water was provided by WaHaHa Company (Hanzhou, China). Instant Ocean sea salt was provided by Tianjin Marine Biological Science Co Ltd of CNSIC (Tianjin China).

### 1.2 Instrumentation

The LC system was a Nanospace SI-2 LC with autosampler (Shiseido Scientific Instruments, Tokyo, Japan). The mass spectrometer used was an AB 3200 Q-Trap (ABSciex, Framingham, MA) equipped with electrospray ionization (ESI), operated in the positive ion mode, and quantification was performed by multiple reaction monitoring (MRM) scan modes. The temperature of the auto-sampler was set at 4°C. The column temperature was 30°C. Chromatographic conditions are shown in Table 1 and MS conditions are listed in Table 2. The compound parameters including the declustering potential (DP), collision energy (CE) cell exit potential (CXP), and the ion source parameters were as follows: curtain gas (CUR), ion spray voltage (IS), temperature, nebulizer gas (GS1), and turbo gas (GS2).

**Table 1:** HPLC conditions of compounds.

Compound (LogP)	solvent A of mobile phase	solvent B of mobile phase	Flow (μL/min)	Column	Mobile-phase gradient
**Ceftazidime** (0.4)	5 mM ammonium formate in water	acetonitrile	300	Shiseido CAPCELL PAK C_18_ MG S5 (2.0 mmi.d.×150 mm)	0–4 min 90%A, 4.1–7 min 10%A, 7.1–13 min 90%A
**Minocycline** (-0.6)	5 mM ammonium formate in water	acetonitrile	300	Shiseido CAPCELL PAK C_18_ MG S5 (2.0 mmi.d.×150 mm)	0–4 min 90%A, 4.1–6 min 20%A, 6.1–11 min 90%A
** Cefotaxime** (-1.4)	5 mM ammonium formate in water	acetonitrile	300	Shiseido CAPCELL PAK C_18_ MG S5 (2.0 mmi.d.×150 mm)	0–4 min 90%A, 4.1–6 min 20%A, 6.1–10 min 90%A
**Netilmicin** (-4.3)	0.1%TFA in water	acetonitrile	200	Shiseido CAPCELL PAK ST (2.0 mmi.d.×150 mm)	0–4 min 85%A, 4.1–6 min 20%A, 6.1–10 min 85%A

**Table 2:** MS conditions of compounds.

Compound	Parameters											
	Compound-dependent							Source-dependent				
	m/z (Da)	ion pairs (Da)	DP^a^ (Volt)		CE^b^ (Volt)	CXP^c^ (Volt)		CUR^d^ (psi)	IS^e^ (Volt)	TEM (℃)	GS1^f^ (psi)	GS2^g^ (psi)
**Ceftazidime**	547.1		27					25	5,500	550	40	60
		→167.2		35		3						
		→468.1		17		7						
**Minocycline**	458.2		46					25	4,000	550	60	60
		→441.2		26		7						
		→283.3		51		4						
		→337.3		60		3						
**Cefotaxime**	456.0		30					25	5,500	550	40	60
		→396.1		8		6						
		→167.1		31		3						
		→324.1		18		4						
**Netilmicin**	476.2		55					10	5,500	400	40	60
		→299.2		27		3						
		→191.5		32		3						
		→160.1		31		3						
		→458.4		12		7						

^a^DP, declustering potential;

^b^CE, collision energy;

^c^CXP, cell exit potential;

^d^CUR, curtain gas;

^e^IS, ion spray voltage;

^f^GS1, nebulizer gas;

^g^GS2, turbo gas.

### 1.3 Sample preparation

Thirty μL of the internal standard (1 ng/mL clenbuterol) was added to 50 μL zebrafish homogenate samples (30 zebrafish were triturated with 100 μL deionized water for 2 min). Protein was removed from samples by vortexing with 200 μL of precipitant for ~2 min. Samples were centrifuged for 10 min at 10,000 rpm, and 10 μL of the supernatant was injected into LC-MS/MS. Protein-precipitates of ceftazidime, cefotaxime, minocycline and netilmicin were acetone, acetone, methanol, and 5% formic acid in water, respectively.

### 1.4 LC-MS/MS method development

To increase the sensitivity of the analytical method, we used HPLC-MS/MS for antibiotic analysis [30,31], as well as an internal standard and a simple protein precipitation step. An LC-MS/MS method for quantifying all compounds in zebrafish was optimized and validated.

#### 1.4.1 HPLC optimization

To control volatility in the LC-MS/MS mobile phase, formic acid and ammonium formate in water were used as mobile phase solvent A and compared. Methanol and acetonitrile were used as mobile phase solvent B and compared. Acetonitrile and 5 mM ammonium formate in water, ceftazidime, cefotaxime, and minocycline had favorable chromatographic behavior, but netilmicin showed less retention in the chromatographic column. To enhance chromatographic retention of netilmicin and avoid ion inhibition, trifluoroacetic acid (0.1%, v/v) was added to the mobile phase.

#### 1.4.2 MS optimization

Compounds were scanned in positive-ion mode. Infusion compound mode was used to optimize compound-dependent parameters (DP, EP, CE, and CXP) with solvent (10 μg/mL) for each analyte. MRM ion pairs were selected by tuning in product ion (MS2) mode. Flow injection analysis (FIA) mode was used to optimize source-dependent parameters (CUR, IS, TEM, GS1, and GS2).

#### 1.4.3 Extraction optimization

Sample extraction for protein precipitation enhanced detection sensitivity, so to optimize the extraction method, methanol, acetonitrile, acetone and formic acid in water were compared.

#### 1.4.4 Method validation

Seven-level standard solutions spiked into blank zebrafish homogenate were used to construct the calibration curves. The linear ranges of ceftazidime, minocycline, cefotaxime and netilmicin were 10–1,000 ng/mL (10, 50,100, 200, 500, and 1,000 ng/mL).

Method precision was evaluated by spiking 50 μL three-point (30, 200, and 1,000 ng/mL) standard solutions into blank zebrafish homogenate. The intra-day precision and accuracy were evaluated by analysis each point of samples three times within one day. The inter-day precision was assessed by repeating the analysis of three concentrations on three consecutive days. Precision at low, medium and high concentrations was expressed as the percentage of relative standard (RSD%), while the accuracy was expressed as mean observed concentration/spiked concentration×100%.

Compound recovery was measured by comparing the peak area of extraction of three different samples (30, 200, and 1,000 ng/mL) to analyte spiked into blank zebrafish homogenate at the same concentration.

The matrix effect was estimated by comparing the peak area of deproteinated samples of blank zebrafish homogenate from three spiked samples (30, 200, and 1,000 ng/mL) with those of the standard samples at equivalent concentrations.

Zebrafish sample were treated as soon as possible, and the short-, long-, and freeze-thaw stability of compounds were measured. For short-term stability, zebrafish samples (30, 200, and 1,000 ng/mL) were kept at 4°C for 24 h before sample preparation. Freeze-thaw stability was investigated after three cycles (one freeze-thaw cycle per day, for three consecutive days) at -20°C for 24 h. Long-term storage stability was measured by assaying zebrafish samples after storage at -20°C for 7 days.

### 1.5 Zebrafish toxicity tests

#### 1.5.1 Ethics Statement

All animal experiments complied with the recommendations in the Regulation for the Management of Laboratory Animals of the Ministry of Science and Technology of China. The protocol was approved by the Committee on the Ethics of Animal Experiments of the Institute of Medicinal Biotechnology, Chinese Academy of Medical Sciences (IMBF20060302). MS-222 (3-aminobenzoic acid ethylester, methanesulfonate salt) was used as an anesthetic for minimization of pain.

#### 1.5.2 Experiment Procedure

Breeding water was artificial sea water prepared by the Instant Ocean sea salt. 20 g of the salt was dissolved in 80 litter reverse osmosis water [32]. Zebrafish wild type (WT) embryos (n = 30) at 6 hpf and larvae (n = 30) at 3 dpf were used. Both embryos and larvae were immersed respectively in 3–4 mL of drug test solutions (drugs were dissolved in breeding water) in a 20-mm dish for 3 days (organogenesis at period of 6–72 hpf), but the solutions for treatment of the larvae were replacement every 24 hours, and zebrafish were observed under a microscope. Abnormal phenotypes and mortality from each treatment group were documented for three sequential observational days. Zebrafish WT embryos (n = 30) incubated in breeding water were controls [17,33]. To remove the resistant effect, zebrafish chorion was torn to allow free distribution of drugs. The embryos (n = 30) at 6 hpf with torn chorions were treated with drug test solutions. Each experiment was repeated three times. Zebrafish body burden of drugs in both the 6 hpf and 3 dpf groups was analyzed separately. The anesthetic was added to the larval embryo solution and then zebrafish bodies were washed three times with breeding water and triturated in 100 μL distilled water on the third day. Values for the 50% teratogenic concentration (TD_50_) and 50% lethal concentration (LD_50_) were calculated using a Bliss algorithm.

## Results

### 2.1 HPLC-MS chromatography

**Fig 1:**
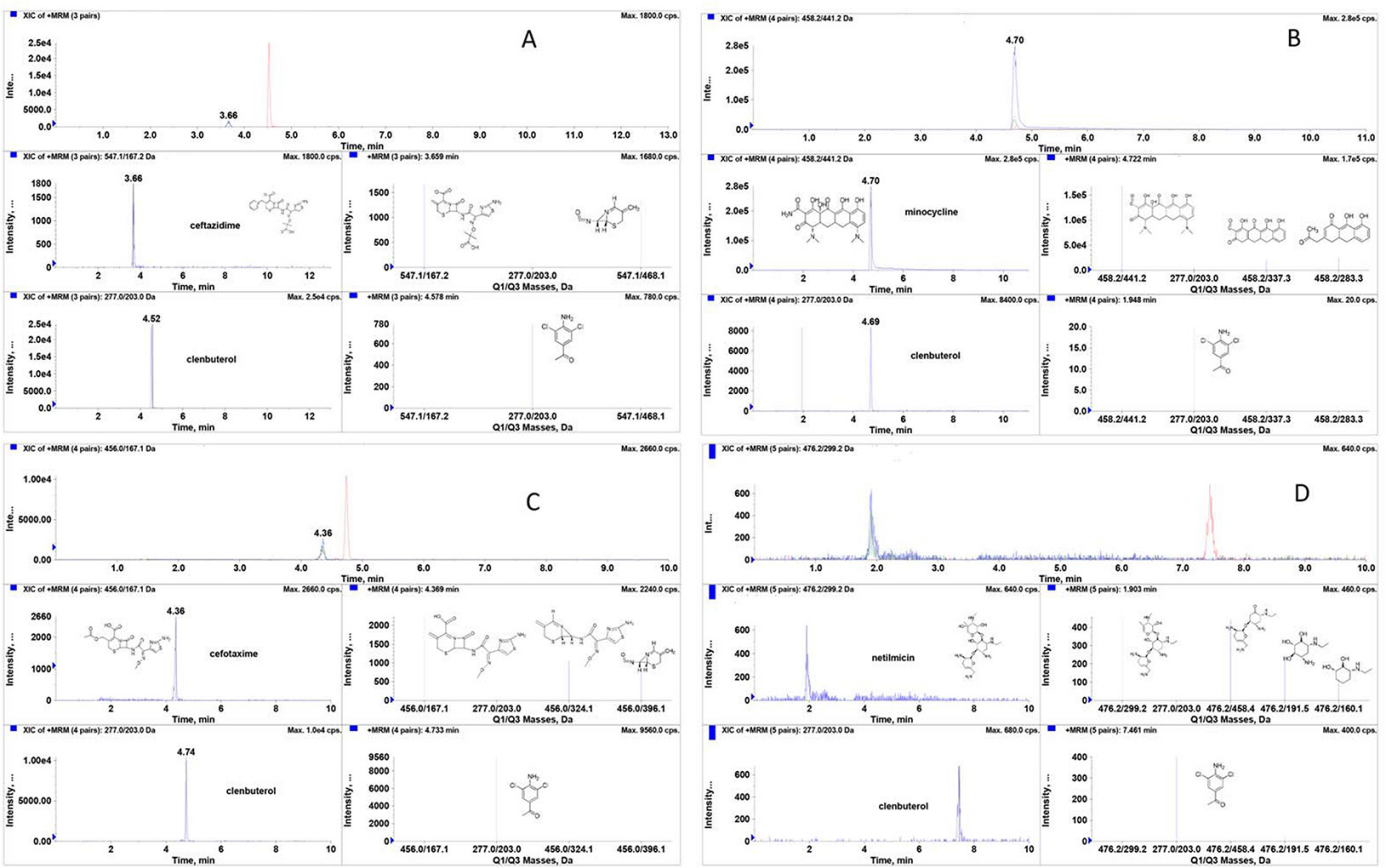
Representative MRM chromatograms of (A) ceftazidime; (B) minocycline; (C) cefotaxime; (D) netilmicin.

Representative MRM chromatograms of ceftazidime, minocycline, cefotaxime and netilmicin are shown in Fig 1. No significant interference was observed at the retention times of the analytes. Fragment ions of ceftazidime, minocycline, cefotaxime and netilmicin were m/z 547.1→167.2→468.1, m/z 458.2→441.2→283.3→337.3, m/z 456.0→396.1→167.1→324.1, and m/z 476.2→299.2→191.5→160.1→458.4, respectively and these data agreed with mass fragmentation pathways [34–36]. Fragment ions of clenbuterol as internal standard were m/z 207→203. Ceftazidime and cefotaxime in acetone had better recovery and minocycline in methanol had better recovery. Netilmicin extracted with 5% formic acid in water was optimal as well (Table 3).

**Table 3:** Extraction conditions of zebrafish sample.

Compound (LogP)		Recovery (%)					
	Solvent	acetone	methanol	acetonitrile	formic acid in water 1, 2, and 5%		
**Ceftazidime**(0.4)	Acetone	82	55	56	—	—	—
**Minocycline**(-0.6)	Methanol	—	86	80	—	—	—
**Cefotaxime**(-1.4)	Acetone	94	56	55	—	—	—
**Netilmicin**(-4.3)	5% formic acid in water	—	—	—	50	69	96

All extractions were via protein precipitation.

### 2.2 Methodology validation

The calibration equations of ceftazidime, minocycline, cefotaxime and netilmicin calculated using linear regression analysis were y = 0.000298x+0.00369, y = 0.0304x+0.0021, y = 0.00152x+0.00104, and y = 0.00114x+0.0118, respectively. The calibration curve was linear over the concentration range of 10–1,000 ng/mL for ceftazidime, minocycline, cefotaxime, and netilmicin. Correlation coefficients (R) were all greater than 0.99. The lower limit of quantification (LLOQ) was set to 10 ng/mL for ceftazidime, minocycline, cefotaxime, and netilmicin, and signal-to-noise ratios (S/N) were all greater than 10.

The intra- and inter-day precision for four compounds was less than 12%. The accuracy for the four analytes was within ±12% and compound recovery exceeded 80%. No significant difference in ion suppression/enhancement (less than 15%) was observed among the compounds at low, medium and high concentrations. The RE of compound stability was lower than 12% when stored at 4°C for 24 h; -20°C for 7 days; and through three freeze-thaw cycles (Table 4).

**Table 4:** Accuracy, precision, recovery, stability, and matrix effect of compounds in zebrafish (n = 3).

Parameter	Drug dose (ng/mL)	Ceftazidime	Minocycline	Cefotaxime	Netilmicin
**Accuracy (%)**	30	98.8	88.1	93.3	101.7
	200	97.6	95.2	98.5	98.0
	1,000	105.8	102.7	95.7	96.6
	30	7.1	3.3	10.7	4.1
**Intra-day Precision (RSD %)**	200	1.0	1.3	2.3	4.2
	1,000	5.5	6.6	8.5	6.3
**Inter-day Precision (RSD %)**	30	9.0	3.7	8.3	7.6
	200	6.3	3.2	5.4	8.2
	1,000	7.4	10.1	7.9	11.6
**Recovery (%)**	30	95.2	80.9	89.1	87.3
	200	87.4	91.0	98.5	92.4
	1,000	90.3	94.5	99.1	99.7
	30	-2.6	3.1	-5.1	-2.8
**Stability (RE %) 4°C for 24 h**	200	-11.5	9.7	2.7	-2.5
	1,000	6.3	5.2	9.1	-2.5
	30	-3.4	-1.1	-2.6	3.7
**Stability (RE %) -20°C for 7days**	200	-6.9	-6.7	-1.2	3.2
	1,000	6.5	2.7	6.2	5.6
	30	-4.3	-8.2	-6.5	-5.5
**Stability (RE %) Three Freeze-thaws**	200	-6.8	2.7	3.7	-7.0
	1,000	9.0	-2.9	4.1	1.1
**Matrix Effect (%)**	30	98.1	85.3	98.7	90.4
	200	84.8	84.9	89.9	85.2
	1,000	109.3	86.3	86.8	105.8

### 2.3 Compound toxicity and absorption

#### 2.3.1 Dose, administration in vivo quantification

**Fig 2:**
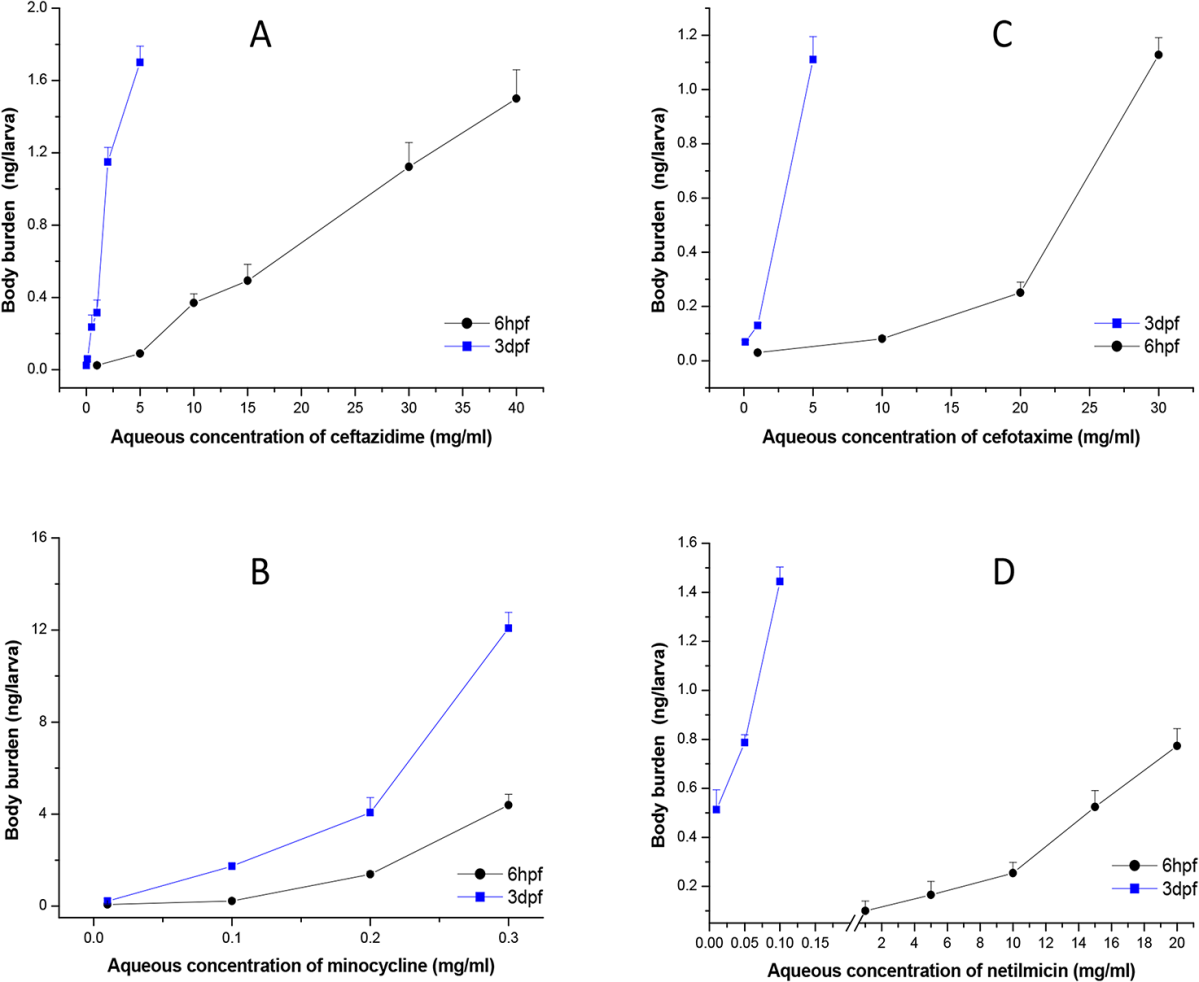
Aqueous concentration and zebrafish body burden of (A) ceftazidime; (B) minocycline; (C) cefotaxime; (D) netilmicin. Each point is the average of triplicate measurements and error bars are standard deviations of the mean. In, ● 6hpf, embryos at 6 hpf were exposed to the drugs for 3 days; ■ 3dpf, larvae at 3 dpf were exposed to the drugs for 3 days.

Compounds were applied to zebrafish at 6–72 hpf (6-hpf treatment group) and at 3–6 dpf (3-dpf treatment group) separately, and the relationships between compound absorption and aqueous doses are depicted in Fig 2.

#### 2.3.2 Toxicity

**Fig 3:**
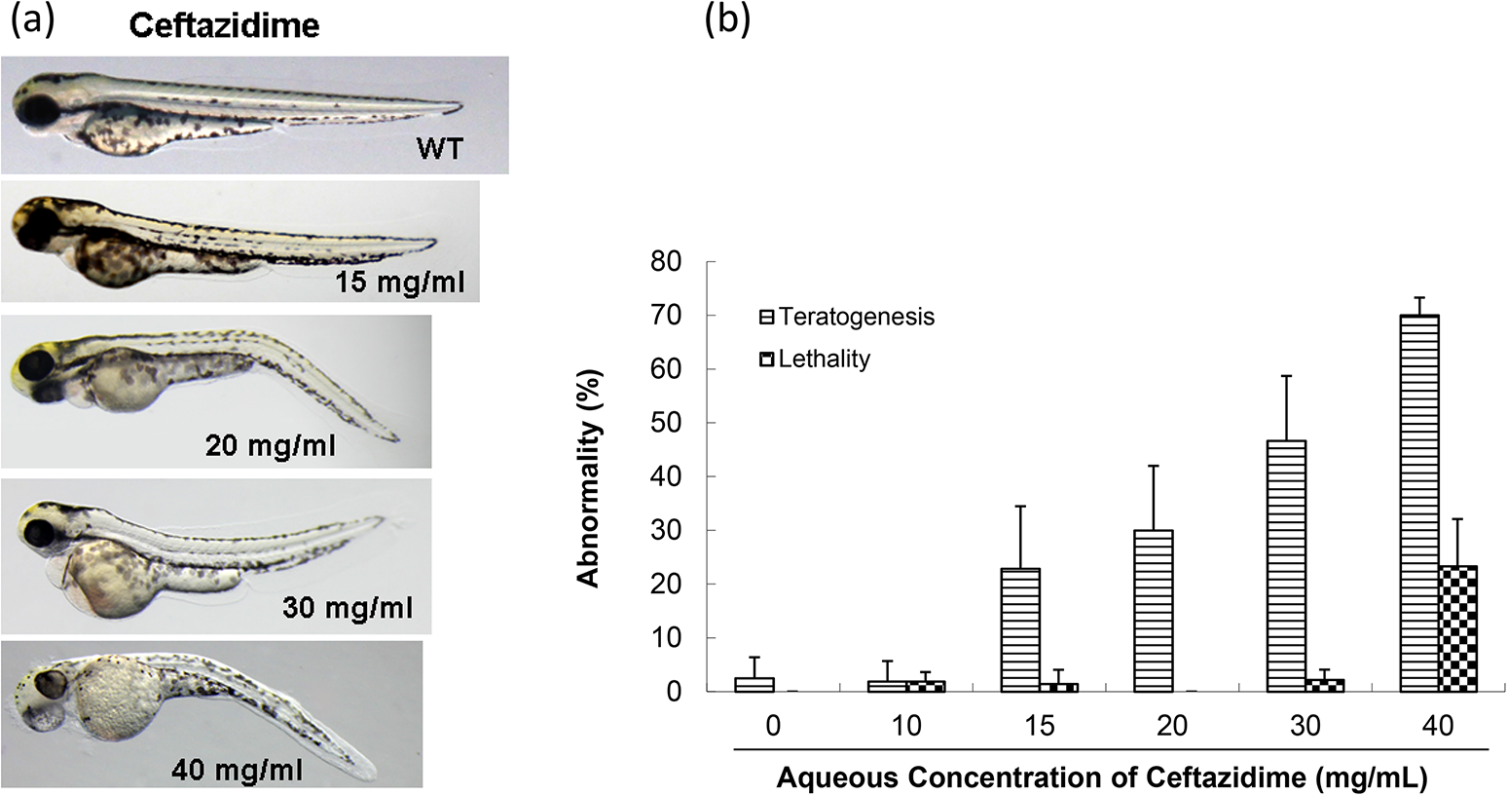
Toxicity of ceftazidime in zebrafish embryo testing. (a) Abnormal phenotypes caused by ceftazidime on zebrafish embryonic development. In, WT, an untreated control; 15, 20, 30, and 40 mg/mL concentrations were used. (b) Comparison of toxicity of ceftazidime in zebrafish embryonic development.

Zebrafish at two developmental periods were observed for three days after exposure to various drug concentrations. At doses not exceeding 10 mg/mL, ceftazidime was not toxic to 6-hpf zebrafish embryos. Abnormal embryos appeared at 15 mg/mL, and increased to 70% at 40 mg/mL (Fig 3A). Teratogenicity manifested as abnormal abdomens, mild blood pooling and congestion and bent, short bodies. After the drug was removed at the hatching stage, abnormal phenotypes were partly recovered. Otherwise, larvae had underdeveloped bladders, dull touch-reactions, and less swimming activity compared to WT larvae (Fig 3B). The TD_50_ for ceftazidime in the 6-hpf group was 28.8 mg/mL and Table 5 lists the LD_50_ values for all tested compounds.

**Table 5:** LD_50_ (Mean±SD) of four compounds with different polarities in zebrafish and rodent.

Drug (LogP)	Zebrafish		Zebrafish (chorion torn)	Rodent^a^	
	6-hpf (mg/mL)	3-dpf (mg/mL)	6-hpf (μg/mL)	Mouse (mg/kg)	Rat (mg/kg)
**Ceftazidime** (0.4)	30.3±3.6	3.9±1.7	7.0±0.8	6,300	6,100
**Minocycline** (-0.6)	0.21±0.2	0.23±0.1	3.0±1.1	154	164
**Cefotaxime** (-1.4)	23.9±3.9	2.1±1.4	6.2±1.5	6,845	7,000
**Netilmicin** (-4.3)	11.2±2.5	0.19±0.1	2.3±1.2	40	25

^a^Rodent data reference [40–43].

**Fig 4:**
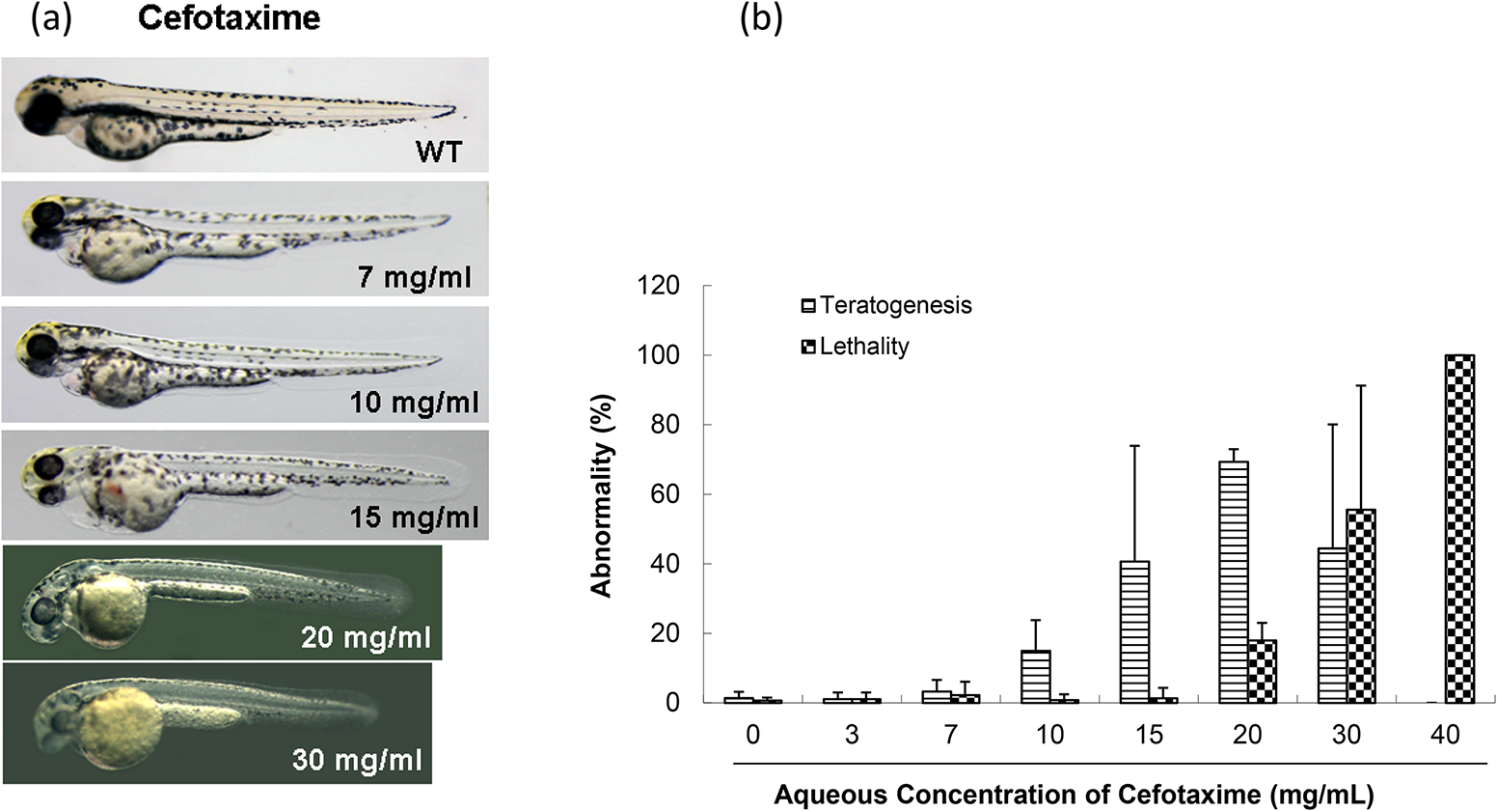
Toxicity of cefotaxime in zebrafish embryo testing. (a) Abnormal phenotypes caused by cefotaxime on zebrafish embryonic development. In, WT, an untreated control; 7, 10, 15, 20, and 30 mg/mL concentrations were used. (b) Comparison of toxicity of cefotaxime in zebrafish embryonic development.

At doses not exceeding 7 mg/mL, cefotaxime had no apparent toxic effect on 6-hpf zebrafish embryos, but teratogenicity increased at doses greater than 10 mg/mL in a dose-dependent manner (Fig 4A), and this was manifested as swollen abdomens and pericardial sacs, deformed heart structures with reduced and congested blood pooling, yolk invagination and short body length (Fig 4B). The TD_50_ for cefotaxime at 6-hpf group was 16.2 mg/mL. Lethality occurred at 20 mg/mL.

No teratogenicity was observed at any dose of minocycline in the 6-hpf group, but all fish died at 0.4 mg/mL.

**Fig 5:**
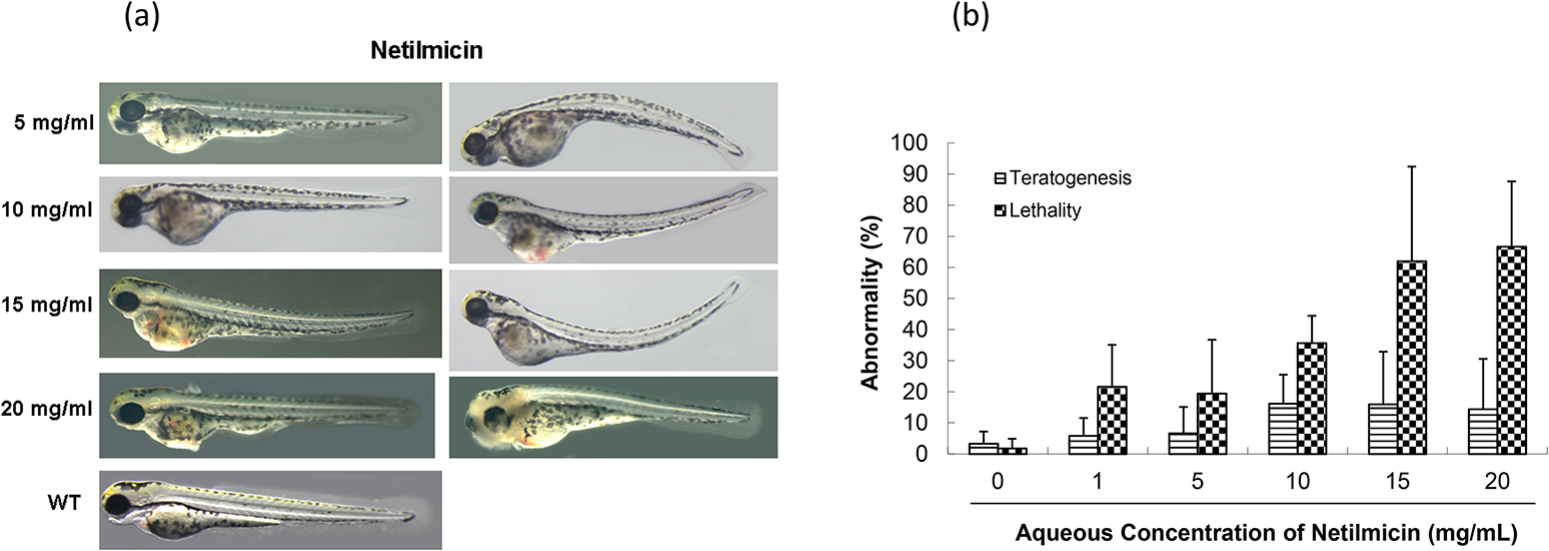
Toxicity of netilmicin in zebrafish embryo testing. (a) Abnormal phenotypes caused by netilmicin on zebrafish embryonic development. In, WT, an untreated control; 5, 10, 15, and 20 mg/mL concentrations were used. (b) Comparison of toxicity of netilmicin in zebrafish embryonic development.

Mild teratogenicity was observed but death in zebrafish increased with increasing doses of netilmicin in the 6-hpf group (Fig 5A). Teratogenicity manifested as bent, enlarged and opaque abdomens with uneven blood clots, deformed heart structures with reduced and congested blood pooling (Fig 5B).

#### 2.3.3 Embryos chorion and toxicity

The LD50 for ceftazidime, cefotaxime and netilmicin on the embryos with torn chorion were all about 4000 times lower than the values obtained from the embryos with chorions, but minocycline was about 100 times lower (Table 5). The results reveal that the differences dependent on the structure of compound, and this partly reflects the ability of compounds to pass through the chorion.

#### 2.3.4 Relationship between drug uptake and toxicity

**Fig 6:**
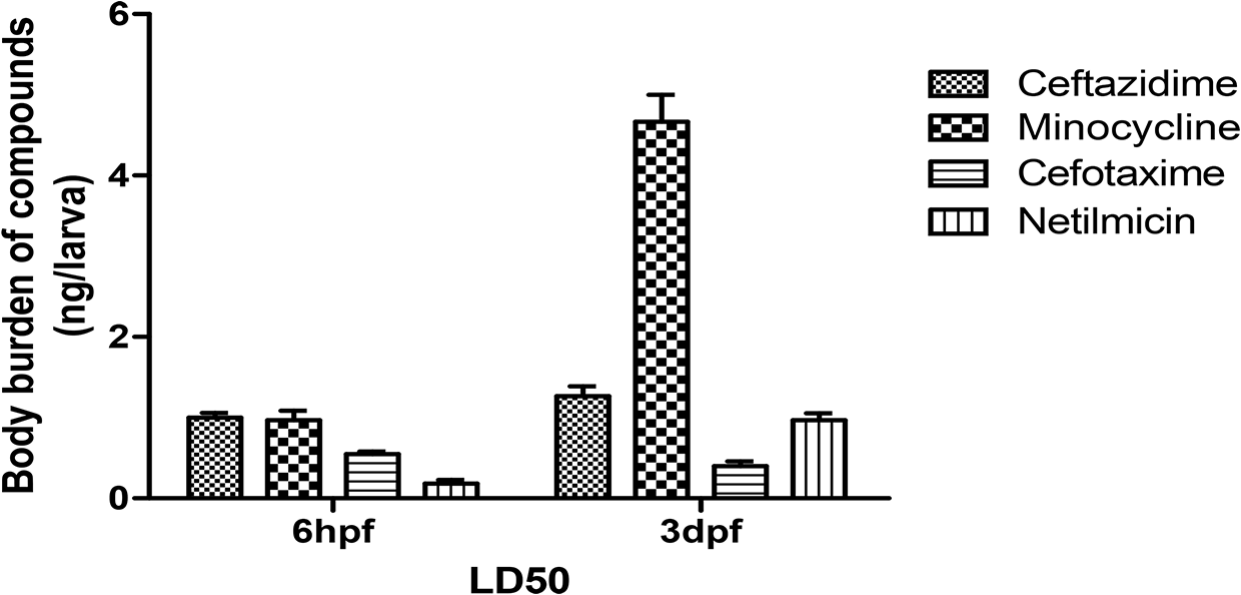
Comparison of uptake of four compounds (LD50) in zebrafish.

Compound uptake was measured in 6-hpf zebrafish and ceftazidime uptake was 0.4 and 1.5 ng/larva [ng/larva = concentration (ng/mL) × 100 μL/30 (zebrafish number)] with 10 and 40 mg/mL treatments. Cefotaxime was 0.1 and 1.2 ng/larva with 10 and 30 mg/mL treatment and minocycline was 0.09 and 4 ng/larva with a treatment of 0.1 and 0.3 mg/mL. Netilmicin was 0.08 and 0.8 ng/larva after treatment with 1 and 20 mg/mL. Non-polar compounds were more absorbed and this likely occurred across the embryonic chorion at 6 hpf. Fig 6 depicts compounds uptake (at LD50) in 6-hpf and 3-dpf groups. These data indicate that the compounds caused real damage independent of absorption effects.

## Discussion

Method validation was completed according to guidelines for validation of bioanalytical methods [37–39]. Calibration curves require correlation coefficients of 0.99 or better. The intra- and inter-day precision values were not to exceed ±15% and stability was considered acceptable if the values were within ±15%. Validation results indicate that the method satisfies requirements for biological sample analysis. HPLC-MS/MS with MRM provided a simple, fast and accurate analysis for quantitative determination of four compounds with different polarities in zebrafish.

Toxicity data show that minocycline in zebrafish embryos is chiefly expressed as lethality, and the abnormal phenotype likely portends death, unlike abnormalities seen with the other compounds.

All four compounds at all doses did not cause teratogenicity in 3-dpf zebrafish. LD_50_ data are in Table 5. Compared with the reported data, the reported intravenous LD_50_ for ceftazidime was ≈6,300 mg/kg for the mouse and 6,100 mg/kg for the rat [40], cefotaxime was ≈6,845 mg/kg for the mouse and 7,000 mg/kg for the rat [41], whereas minocycline was ≈154 mg/kg for the mouse and 164 mg/kg for the rat [42], and netilmicin was ≈40 mg/kg for the mouse and 25 mg/kg for the rat [43]. These data had similar trends with those observations made in 3-dpf zebrafish.

Comparing drug uptake to concentration administered, 3-dpf zebrafish absorbed more drug. Aqueous compounds are mainly absorbed in the embryo through the embryonic chorion but drugs can be absorbed through the skin and via swallowing at larval stages beyond 3 dpf. Therefore, the zebrafish development stage is critical when selecting this species for toxicity testing. We concluded that zebrafish embryos at 6 hpf are more sensitive for evaluating toxicity on embryonic development because the main organs of begin developing from ≈12 hpf [44], However, to assess chemical acute toxicity in mammal adults, larvae at 3 dpf are more suitable.

Fig 6 depicts the relationship between compound structure and toxicity in zebrafish by removing the absorption effect. More drug uptake was inversely proportional to toxicity. Drug uptake (at LD_50_) for ceftazidime (LogP 0.4), minocycline (LogP -0.6), cefotaxime (LogP -1.4), and netilmicin (LogP -4.3) in 3-dpf groups were ≈1.3, 5.6, 0.5, and 0.9 ng/larva, respectively, and in 6 hpf zebrafish were ≈1.1, 1.2, 0.8 and 0.4 ng/larva, respectively. Polar compounds were more toxic and LD_50_ values for minocycline were similar for both zebrafish groups but minocycline was more absorbed in 3-dpf animals (5x greater) than in the 6-hpf group. Thus, the 3-dpf embryo is more tolerant to minocycline.

It appears that ceftazidime, cefotaxime and netilmicin all caused cardiotoxicity in the zebrafish when be treated in developmental period. Although there are no serious adverse effect reports of those antibiotics on cardiotoxicity in clinical use, Czeizel et al. also reported that treatment with some cephalosporins during pregnancy does not seem to present a detectable teratogenic risk to the fetus using pair analysis of cases [45]. However, as drug safety is substantially more difficult to demonstrate than its efficacy, and sex differences exist in distribution volumes, transport proteins, and drug clearance, even pregnancy itself affects the absorption, distribution, metabolism, and elimination of a drug, it is necessary to improve our understanding of the use, safety, and efficacy of drugs for the treatment of women and the fetus during pregnancy [46]. Alternative strategies need to be developed to characterize the safety information of drugs. The results obtained by zebrafish toxicity tests may give us extra knowledge on the antibiotics.

## Conclusions

In conclusion, a rapid, sensitive and reliable LC-MS/MS method using MRM for compounds with different polarities was established for measuring compounds absorbed in zebrafish, and this method was successfully applied to evaluate compounds in zebrafish toxicity tests. Zebrafish embryos absorbed compounds uniquely depending on the developmental period and different with respect to development. The 6-hpf embryos can be used to evaluate toxicity during embryonic development, and larvae at 3 dpf can be used to mimic acute toxicity in adults.
